# Torsion of an Accessory Hepatic Lobe with Embedded Gallbladder: In an 11-Month-Old Boy

**DOI:** 10.1055/s-0037-1607218

**Published:** 2017-10-15

**Authors:** Chiman Lal Thakral, Ganji Shivalingam, Faizan Manzoor Dar, Nimish Thakral

**Affiliations:** 1 Department of Paediatric Surgery, Royal Hospital, Muscat, Oman; 2 Department of Pediatrics, Sohar Hospital, Sohar, Al Batinah, Oman; 3 Department of Surgery, Sohar Hospital, Sohar, Al Batinah, Oman; 4 Department of Surgery, Shri BM Patil Medical College, Bijapur, Karnataka, India

**Keywords:** accessory hepatic lobe, torsion of accessory liver lobe, omphalocele

## Abstract

An accessory lobe of the liver is a rare entity in clinical practice which is diagnosed incidentally. Infrequently, it may present as torsion with a clinical picture of an acute abdomen, a palpable mass, and may be associated with liver function abnormalities. Many of these patients have a history of previous surgery for congenital abdominal wall defects such as omphalocele. We present an extremely rare case of torsion of an accessory hepatic lobe in an 11-month-old male patient who presented in a state of shock. The infant underwent laparotomy and excision of the accessory lobe. Here, we aim to emphasize the importance of prompt management and early resection which is the cornerstone of a favorable outcome.

## Introduction

Cases of congenital accessory hepatic lobe torsion (AHL) are largely unheard of with only 22 reported cases in children, out of which there were only 7 reported in infants, in English literature as of 2017. 1

AHL may be sessile with a wide base of continuous hepatic parenchyma connected to the liver proper, appearing as small tongue-like projections from the surface of the liver (i.e., Riedel's lobe) or rarely exist as a pedunculated mass of hepatic parenchyma attached to a vascular pedicle. 2 Most of the cases are asymptomatic. When pedunculated, AHL can undergo torsion resulting in ischemia, which can then present as an acute abdomen. To help identify and properly treat such cases we aim to provide more insight on the subject.

We present a case of torsion of the AHL with an embedded gallbladder, embedded in it, in an infant.

## Case Report

An 11-month-old boy weighing 8 kg presented to the emergency department with severe pallor, cyanosis, and signs of respiratory distress. He was a known *G6PD* -deficient and had a history of omphalocele repair in the neonatal period. On examination, he was in distress, mildly cyanotic, afebrile, and not jaundiced. His vital signs were: heart rate (HR): 158 bpm, respiration rate: 40 bpm, blood pressure: 85/40 mm Hg, and temperature: 35.3°C. The examination of the respiratory system showed the presence of doubtful bilateral basal crepitations.

On abdominal examination, there was soft distention with a palpable mass occupying the right half of the abdomen, extending to the umbilicus, which was tender and there was no blood or mucus on per rectal examination. Laboratory investigations revealed a white cell count of 36 × 10 ^3^ /µL, hemoglobin: 9.7 g/dL, serum sodium, potassium, and creatinine levels were normal, the alanine aminotransferase (ALT) value was 48 U/L, serum bilirubin, and alkaline phosphatase levels were normal. The C-reactive protein was normal, and the venous blood gas analysis showed a pH of 7.1, P o_2_ : 36%, P co_2_ : 73%, HCO _3_ : 22.7, and base excess of −7.5. Within 3 hours the patient became paler, and the hemoglobin dropped to 6 g/dL. He subsequently received an urgent blood transfusion and was started on antibiotics; chest X-ray was unremarkable.

On abdominal ultrasound, a 10 × 8 cm mass was noted. Evidence of marked ascites was present, and the gallbladder was distended.

**Fig. 1:**
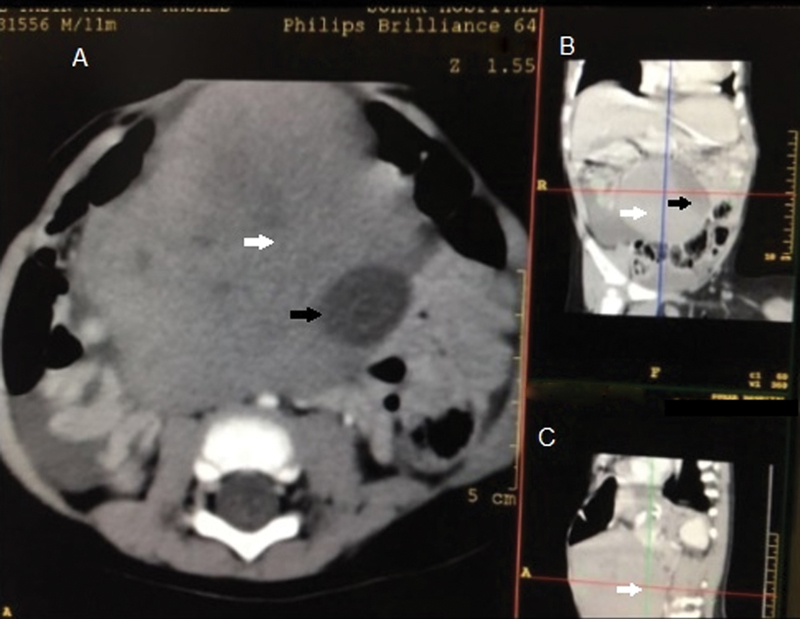
( **A** – **C** ) White arrows show the accessory hepatic lobe and the black arrows show the embedded gallbladder.

A computed tomography scan of the abdomen with oral and intravenous contrast confirmed a large high density (100 HU) midline mass measuring 7.8 × 8.9 × 9.4 cm ( Fig. 1A–C ).

This gave the initial impression of a volvulus involving a lobe of the liver and gallbladder or internal herniation. Moderate amount of free fluid was noted in the subhepatic, right paracolic gutter, and the pelvis.

**Fig. 2:**
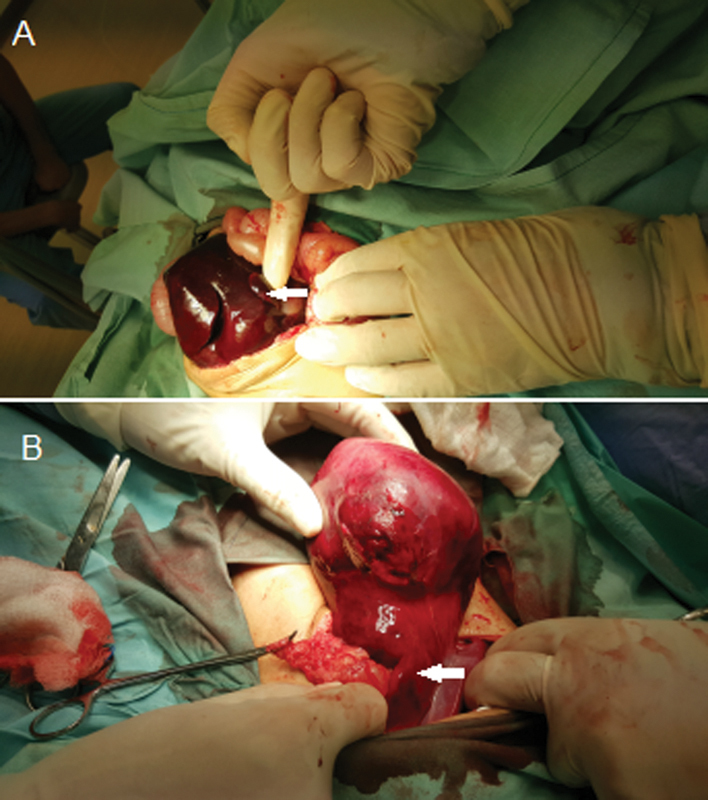
Operative pictures: ( **A** ) Dusky Congested accessory liver lobe with the congested gallbladder (white arrow) embedded in it. ( **B** ) The liver lobe regaining normal color after untwisting the pedicle (white arrow).

An urgent exploratory laparotomy was then performed through an upper transverse abdominal incision. We discovered a severely congested hepatic lobe with the gallbladder in situ. Due to the presence of the gallbladder, we presumed that this was the right lobe of the liver ( Fig. 2A ).

This was found to be under torsion on an abnormally long pedicle. Also, a lobe of the normal liver was found in the location of the right lobe without the gallbladder attached to it. Following this observation, the lobe was untwisted, and signs of reperfusion were noted. The bowel was normal in color, the duodenojejunal flexure was located normally, but the cecum was mobile ( Fig. 2B ).

On the postoperative day 1, the patient showed signs of recovery inferred by his improving blood gases and vital signs, but 24 hours later he again suddenly became pale, tachycardiac with an HR of 185 bpm, and a concurrent drop in hemoglobin from 10 to 6 g/dL. The abdomen became tense again, and an urgent ultrasonography was done but was inconclusive regarding blood flow to the affected part of the liver. Liver function tests revealed marked elevation of ALT level up to 3,854 U/L. An urgent relaparotomy was performed, and the previously torted hepatic lobe was found to be under torsion again; with a dusky hue and patchy necrosis, therefore excision of the ischemic torted AHL along with the gallbladder was performed. The remaining lobes of the liver were noted to have an independent pedicle containing the portal triad. The patient was given blood products and was continued on antibiotics. On ultrasound 1 after relaparotomy, the liver had normal size and a uniform texture with normal hepatic and portal veins. There was no evidence of thrombus formation, and the common bile duct and intrahepatic biliary radicles were not dilated. The patient gradually recovered and was discharged in good condition 12 days after admission. He remained asymptomatic on follow-up after 14 months.

## Discussion

The AHL by definition is a developmental congenital anomaly of the hepatic bud that arises from the endodermal caudal foregut. 3 There are differing ways to classify this entity, 4 5 largely it is classified based largely on the size (10–30 g) and location of the lobe (attached to the liver or ectopic) or the presence of a capsule and biliary drainage system. 3 The viability is assessed by the presence of the portal triad in the AHL. 5

Similar to previously reported cases, the diagnosis of the AHL torsion was only definitively made intraoperatively. As of 2017, only 22 cases have been reported in children regarding this anomaly with 7 cases reported in infancy. 1 There seems to be a male predominance of AHL in infancy whereas in females AHL was often an incidental finding. The reason for this phenomenon remains unknown. 1 4 Imaging techniques without a high index of suspicion make the preoperative diagnosis difficult. In this case, due to the history of an omphalocele and the presentation of an acute abdomen and a palpable mass, there was reasonable indication to suspect AHL pathology. 3

Our patient presented in a very sick state probably as the extent of torsion was severe, and the gallbladder was included in the torted lobe. Depending on the location and duration of torsion of the AHL, it can present with various symptoms. A pedunculated AHL carries a higher risk of torsion than the other types of AHLs. Moreover, severe congestion and insufficient blood supply may lead to rupture of larger vessels. 2 Strangulation of the vascular supply to the liver due to the twisting of the mesentery of a large AHL may lead to hepatic ischemia necessitating transplantation. 6 However, our patient had a successful outcome, like another case, 7 which is attributed to the prompt recognition and the surgical intervention performed.

The torted AHL in our patient was not necrotic and had regained color upon untwisting the lobe, yet it underwent torsion again and had to be excised the following day. It can be postulated that an excision is a good option as the primary approach instead of trying to preserve any seemingly viable liver tissue after it has been untwisted. However, we cannot rule out that fixation of the untwisted lobe would have been successful too in our case.

## Conclusion

Without a high index of suspicion, the diagnosis of an AHL is difficult before surgical exploration. When discovered, the excision during initial laparotomy is a good option.

## New Insights and the Importance for the Pediatric Surgeon

Torsion of the accessory hepatic lobe torsion is an extremely rare condition and has the potential to cause serious complications. Pediatric surgeons must be aware of this entity while managing cases of acute abdominal pain, particularly those with a history of omphalocele repair.
